# Ultrasound-Guided Electroacupuncture for Thoracic Myofascial Pain Syndrome: A Case Report

**DOI:** 10.7759/cureus.36973

**Published:** 2023-03-31

**Authors:** José Afonso, Tânia Carvalho, Letícia Cruz, Helder Cardoso

**Affiliations:** 1 Anesthesiology, Centro Hospitalar Tâmega e Sousa EPE, Penafiel, PRT

**Keywords:** case report, safety, ultrasonography, acupuncture, myofascial pain syndrome

## Abstract

Myofascial pain syndrome is a painful condition characterized by trigger points in muscles that can be treated effectively with acupuncture. While cross-fiber palpation can help localize trigger points, needle accuracy may be limited and accidental puncture of delicate structures, such as the lung, is a risk, as evidenced by reports of pneumothorax after acupuncture. Ultrasound imaging can help in reducing the risk of iatrogenic pneumothorax from needling, but there is a paucity of papers describing the use of ultrasound imaging during acupuncture. We present a report on electroacupuncture treatment for myofascial pain syndrome using real-time ultrasound guidance, aimed at avoiding accidental puncture of the pleura when targeting deep muscle layers in the thoracic region.

## Introduction

Acupuncture involves the insertion of thin metallic needles at specific points in the body. Western medical acupuncture is a modified version of traditional Chinese acupuncture that incorporates a current understanding of anatomy, physiology, pathology, and evidence-based medicine principles [1]. Practitioners of Western medical acupuncture tend to focus less on specific points along Chinese meridians and instead choose points based on current concepts of disease mechanisms. Electroacupuncture uses an electric current generator that sends pulses of electrical current through acupuncture needles inserted in the body. Several publications have evaluated the efficacy of acupuncture techniques for myofascial pain syndrome (MPS), which have shown to relieve pain and increase the range of motion [2-4]. Although acupuncture is generally safe, it is not completely risk-free. Complications such as lung perforation and pneumothorax have been reported [5].

Ultrasound guidance may reduce the risk of accidental puncture of vital structures during conventional drug injections when used by experienced operators. Furthermore, ultrasound provides a live view of internal structures, which allows for precise navigation toward intended targets, even in obese patients. In our pain clinic, after localizing a trigger point (TrP) by palpation, we often observe various layers of muscle and fascia in the ultrasound image beneath a thick layer of subcutaneous fat. Besides, in the thoracic region, the pleura is frequently located dangerously close to the treatment site.

To treat MPS in the thoracic region, we used ultrasound-guided electroacupuncture. Our case report does not focus on the efficacy of acupuncture but rather on how to improve accuracy and safety during needle insertion.

## Case presentation

**Figure 1:**
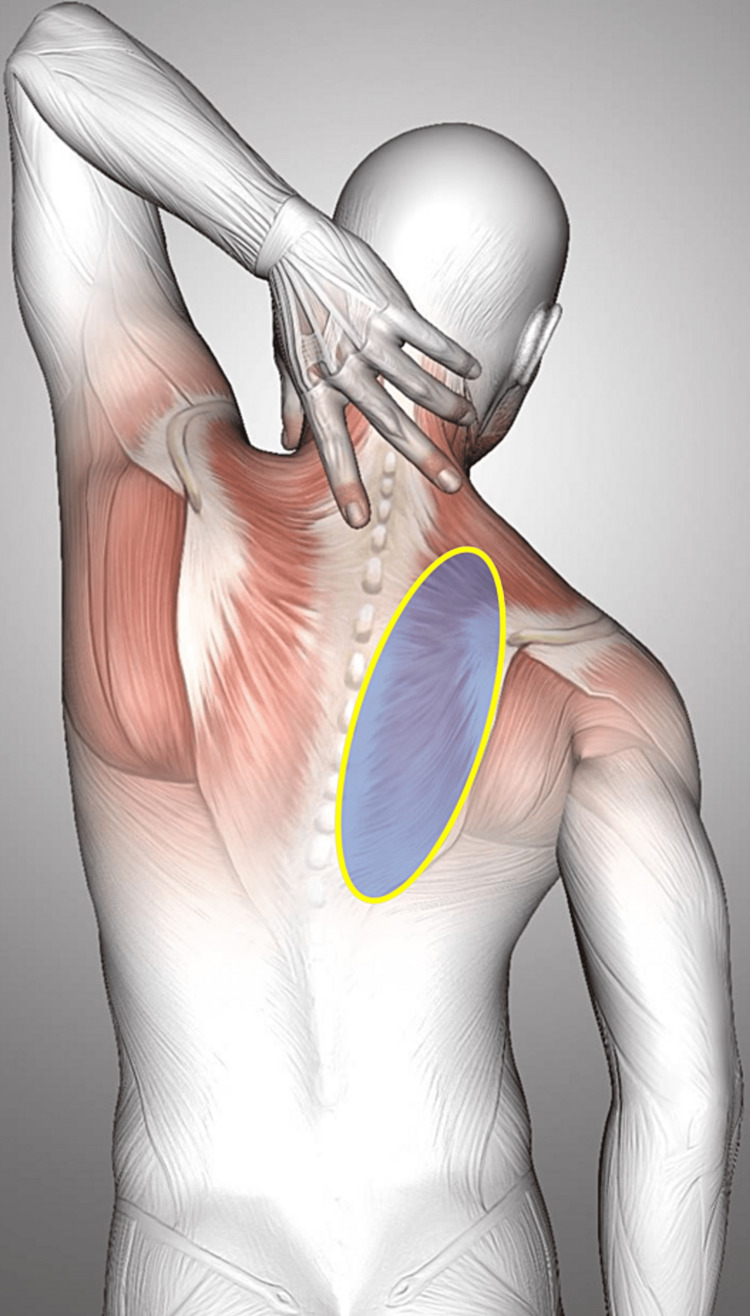
Painful area. The yellow line limits the painful area reported by the patient. Image by Kjpargeter on Freepik. Edited by Helder Cardoso.

A 56-year-old male presented to our chronic pain department complaining of pain localized in the posterior thorax just medial to the right scapular margin. He described moderate to intense pain (numeric pain scale = 7) and a limited range of motion of the ipsilateral shoulder for more than five years (Figure 1).

The patient also complained of frequent sleep disturbances and a depressed mood. He was obese (BMI = 33 kg/m^2^) and was taking medication for hypertension and depression. No other illnesses were reported. He had already been treated with non-steroidal analgesics, muscle relaxants, and physical therapy, but reported low or temporary efficacy.

Upon examination, he experienced parascapular spontaneous pain and tenderness that was aggravated by active movement of the shoulder and spread to the ipsilateral neck. It was possible to palpate a discrete taut muscle band under a thick subcutaneous layer; however, the classic twitch response was not clearly discernible. Chest radiography and ultrasonography of the painful region did not reveal any abnormalities. Laboratory test results were normal, including serum calcium, magnesium, vitamin D, and thyroid hormone levels.

Based on history and physical examination, it was concluded that the patient was diagnosed with chronic MPS, with TrPs in the parascapular region as the underlying cause, although the typical muscle twitch response was not observable.

**Figure 2:**
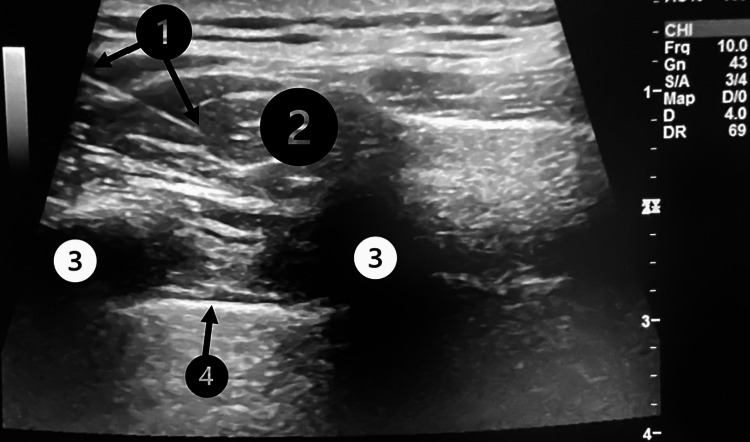
Real ultrasound image obtained during one treatment session The top of the image corresponds to subcutaneous fat. The bottom corresponds to the lung. 1) The acupuncture needle shaft. 2) Target muscle; 3) Ribs; 4) Pleura. Image obtained by Pedro Afonso and edited by Helder Cardoso, with patient consent.

The reported insomnia and altered mood led us to choose acupuncture instead of conventional TrP deactivation. In this case, a six-session weekly electroacupuncture protocol was chosen. The target area for treatment included the muscle layers along the medial border of the scapula, specifically the trapezius, rhomboid, and erector spinae muscles. The pleural membranes and lungs were very close to the target area. To ensure accurate targeting in the superficial and deep muscle layers, below the thick subcutaneous layer, but safely away from the pleural membrane to prevent pneumothorax, we decided to guide the acupuncture needles with real-time ultrasound imaging (Logic-e portable machine, linear probe, 10 MHz; GE Healthcare, Chicago, IL) (Figure 2).

After informed consent was obtained, eight acupuncture needles were placed every session along and parallel to the palpable painful and taut band. The electroacupuncture settings were 4 Hz and 100 ms for a total duration of 20 min, with an intensity considered significant but tolerable by the patient (Electric Current Generator: ES-160, ITO, Japan).

One month after completing the treatment, the patient reported a reduction in the numeric pain scale from 7 to 3, improved range of motion, and better sleep. The mood did not improve. No complications were reported.

## Discussion

MPS is associated with the presence of TrPs within muscles. It is one of the most common musculoskeletal chronic pain syndromes [6]. TrPs are defined as spots of intense tenderness and hyperirritability in muscles localized in taut, palpable bands, which mediate a local twitch response upon palpation.

The pathophysiology of TrPs is not completely understood, and many theories have been proposed to explain their mechanisms [7]. One hypothesis suggests that TrPs are due to dysfunction of the neuromuscular junction. Another hypothesis is the integrated trigger hypothesis, which describes a sequence of events initiated by local repetitive microtrauma, which leads to a self-perpetuating ischemic contracture of muscle fibers [8]. Recent studies have suggested another mechanism, which involves central sensitization and neurogenic inflammation. This could cause the formation of hyperirritable myofascial TrPs in the absence of regional muscle injury [9]. Common predisposing factors for the development of MPS include trauma, deconditioning, abnormal posture, repetitive mechanical stress, psychological stressors, structural diseases such as scoliosis, poor sleep quality, and systemic disorders such as vitamin deficiencies or hypothyroidism [6].

The gold standard treatment for MPS is TrP injection [10]. It has been shown to be one of the most effective modalities for inactivating TrPs and providing immediate relief of symptoms [10]. It is a safe procedure when used by clinicians with appropriate training. Common substances injected are local anesthetics, saline, corticosteroids, and botulinum toxin type A. However, several studies also indicate that dry needling, which involves perforation of TrPs without injection of drugs, may be as effective as the injection of a local anesthetic, which would explain why such a precise localization of the penetration is required [10]. Accurate needle placement in a myofascial TrP is vital to improve efficacy and prevent complications of TrP injection [11]. Acupuncture and electroacupuncture are also effective treatment options [2, 3, 12]. Acupuncture is indeed a form of “dry needling”, provided that needles are inserted in the TrPs. Most acupuncture therapies showed superiority over other single physical therapies in terms of pain decrease and physical function improvement [12]. Moreover, acupuncture benefits patients in terms of local and specific treatment of TrPs but may also provide beneficial systemic effects on mood, anxiety, insomnia, fatigue, and global wellness [2].

Regarding the use of ultrasound in guiding acupuncture needles, Leow et al. described the use of ultrasound on a volunteer team member by inserting a needle after mapping the site (no real-time ultrasound treatment was used). They highlighted the potential usefulness of ultrasound in acupuncture and as a model to standardize treatments [13].

Our case report is not about the efficacy of acupuncture but about how to address specific issues frequently encountered in clinical practice. One frequent problem encountered during needling therapies is the difficulty in determining the appropriate depth when introducing needles into regions with multiple muscle layers. Taut bands are not necessarily the most superficial muscle layers; therefore, correct positioning in deeper layers can be difficult to perform accurately and safely, and obese patients are a particular challenge. TrPs injection or needling in the thoracic or abdominal regions has an inherent risk of accidental puncture of internal organs. In addition, it may be difficult to maintain a consistent needle depth between treatment sessions using blind techniques guided only by anatomical references or palpation. Our report describes the use of ultrasound to address these issues. Under real-time imaging guidance, it was possible to distribute the needle tips below the thick subcutaneous fat through different muscle layers (superficial and deep) and to avoid accidental puncture of the pleura. It was possible to pursue consistency in the needle tip positions between the various sessions. Unfortunately, ultrasound has limitations in identifying TrPs within muscles. To increase the probability of correctly needling the TrPs, the needles were placed in the superficial and deep muscle layers, at the points where the pain was most intense upon palpation.

The decision to choose six-session weekly electroacupuncture as a therapeutic option over conventional TrP injection deactivation was reasonable because it took into consideration the potential benefits of electroacupuncture in treating depressed mood and insomnia.

**Figure 3:**
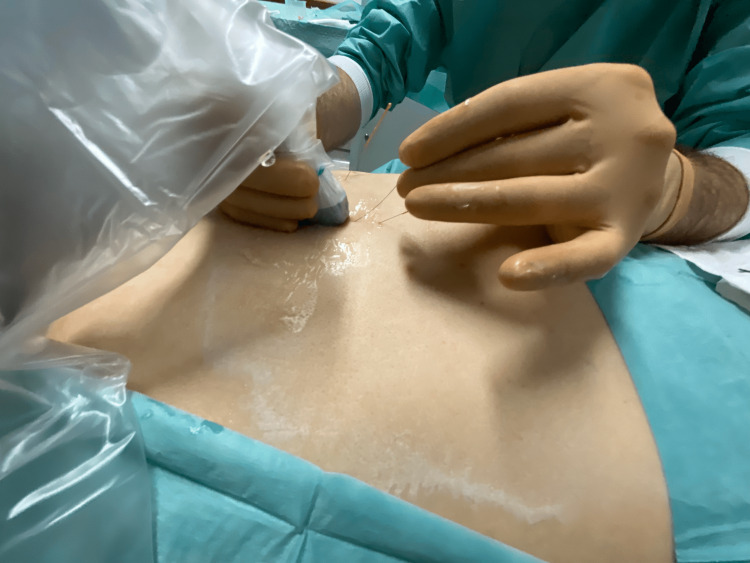
Needle placement under ultrasound guidance Insertion of a needle in the right thoracic parascapular region. The ultrasound probe is protected by a plastic sleeve and sterilized ultrasound gel was used. Image obtained by Pedro Afonso and edited by Helder Cardoso, with patient consent.

**Figure 4:**
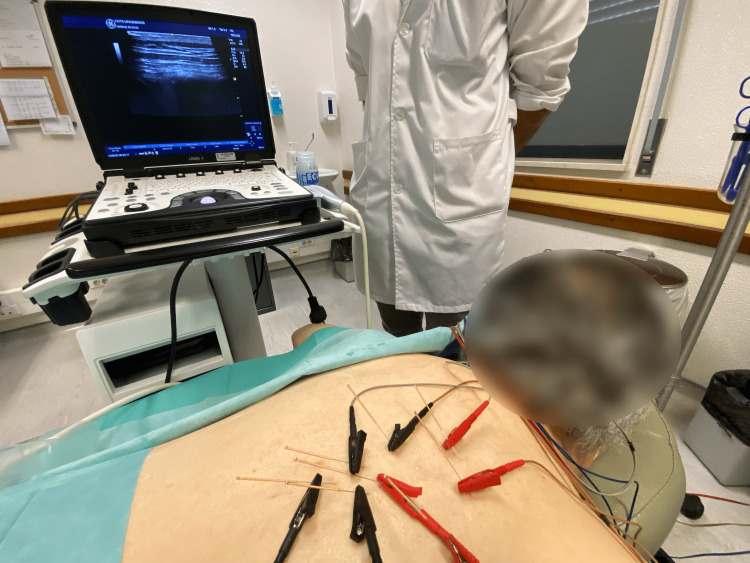
Final setup Image obtained and edited by Helder Cardoso, with patient consent.

The limitations we found were the slight difficulty caused by the ultrasound gel during needle placement, the difficulty in manipulating the ultrasound probe after some needles were already in place, and the slightly increased difficulty in visualizing the thin needle shafts owing to their reduced thickness. In addition, the time it took to perform the procedure was longer, and the cost of the procedure was higher than that of the usual blind technique. However, we believe that the technique is simple for a pain management specialist to perform with ultrasound training (Figures 3, 4).

## Conclusions

This case report describes the successful use of ultrasound-guided electroacupuncture for the treatment of myofascial TrPs in the thoracic region, aiming to optimize needle placement accuracy and avoid accidental pleural perforation. Although acupuncture is generally safe, complications such as lung perforation and pneumothorax have been reported. The use of ultrasound-guided acupuncture, in this case, resulted in significant pain relief without any complications, indicating that it may be a useful technique for practitioners of Western medical acupuncture when treating TrPs in the thoracic region. However, despite the satisfactory results, there is a need for further clinical studies to evaluate the cost-effectiveness, safety, and efficacy of ultrasound-guided acupuncture compared to conventional blind techniques.
